# DNA methylation profile of a hepatosplenic gamma/delta T-cell lymphoma patient associated with response to interferon-α therapy

**DOI:** 10.1038/s41423-020-0518-4

**Published:** 2020-08-20

**Authors:** Jaydeep Bhat, Anke K. Bergmann, Silvio Waschina, Christoph Nerl, Christoph Kaleta, Reiner Siebert, Ole Ammerpohl, Dieter Kabelitz

**Affiliations:** 1 Institute of Immunology, Christian-Albrechts-University Kiel & University Hospital Schleswig-Holstein, Campus Kiel, Kiel, Germany; 2 Metabolic Programming, School of Life Sciences Weihenstephan, Technical University Munich (TUM), 85354 Freising, Germany; 3 Institute of Human Genetics, Christian-Albrechts-University Kiel & University Hospital Schleswig-Holstein, Campus Kiel, Kiel, Germany; 4 Institute of Human Genetics, Medical School Hannover, Hannover, Germany; 5 Institute of Experimental Medicine, Christian-Albrechts-University Kiel & University Hospital Schleswig-Holstein, Campus Kiel, Kiel, Germany; 6 Institute of Human Nutrition and Food Science, Christian-Albrechts-University Kiel, Kiel, Germany; 7 Städtisches Krankenhaus München-Schwabing, Munich, Germany; 8 Institute of Human Genetics, Ulm University & Ulm University Medical Center, 89081 Ulm, Germany

**Keywords:** Lymphoma, Oncology

The 2–5% γδ T cells in healthy human peripheral blood recognize nonpeptide antigens, in contrast to αβ T cells.^1^ Distinct γδ T-cell malignancies have been identified, with hepatosplenic T-cell lymphoma (γδ-HSTL) being a rare but aggressive (median survival 13 months) subset.^2^ γδ-HSTL usually presents with hepatosplenomegaly without lymphadenopathy, anemia, and thrombocytopenia. It is histologically characterized by the involvement of the hepatic sinusoids, splenic red pulp and bone marrow interstitium, with or without leukemic presentation in peripheral blood.^3^ A standard treatment regimen is not well established, but therapy mostly includes various chemotherapeutic agents. Notably, interferon-α (IFN-α) has been proven to be effective in some patients with γδ-HSTL, but other patients require allogeneic or autologous stem cell transplantation.^4^

Here, we present a longitudinal comprehensive DNA methylation analysis of a patient with γδ-HSTL who reached complete remission on continuous therapy with interferon-α2c (IFNα2c). The 39-year-old female patient presented with chronic lymphocytosis (WBC count 22,300/µl, with 98% lymphocytes, 93% of those with the γδ T-cell clonal phenotype), mild hepatosplenomegaly, severe anemia (hemoglobin (Hb) level 8.9 g/dL, haptoglobin level <5 mg/dl), LDH 300 U/ml and distinct Coombs-positive hemolysis. No peripheral, intrathoracic, or abdominal lymph node enlargements were recorded, but the skin showed multiple vitiligo-like lesions with lymphocyte infiltration. Bone marrow infiltration was also present. The expanded circulating lymphocytes resembled large granular lymphocytes (LGLs) with TCRγδ surface expression.^5–7^ The patient was treated with IFNα2c (10^6^ IU s.c.) over a period of 6 years. During the first two years of treatment, an increase in the Hb level (from 8.9 to 11.2 g/dL) and a steady decrease in the leukocyte count (from 22,300/µl to 7200/µl) were observed. The hematological parameters and peripheral blood mononuclear cell (PBMC) phenotyping were identical before and after the visit at which IFNα2c treatment was initiated^5^ (Supplementary Table S1).

In this retrospective study using frozen PBMCs collected from this patient at five time points (hereafter referred to as visits 1–5) over 22 months after the initiation of IFNα2c therapy, DNA methylation analysis was performed using Infinium 450k BeadChip arrays (see Supplementary Material). After removing all measurements with detection *p* values > 0.001, we applied a variance filter (σ/σ_max_ > 0.01) to focus on loci showing the strongest changes (*n* = 12,825 loci). After performing a Pearson correlation analysis (day of treatment ~ avg. beta value), we included only CpG loci that showed a significant (FDR < 0.05; Benjamini–Hochberg) and continuous change in DNA methylation under treatment (day 54–652). Of the resulting 273 loci, 49 showed an increase in methylation (Pearson correlation coefficient range: 0.9908–0.9990), while the remaining loci showed a negative correlation (Pearson correlation coefficient range: −0.9907 to −0.9916) (Supplementary Table S2).

In the exploratory analysis, we observed statistically significant (paired Wilcoxon test, FDR < 0.05; Benjamini–Hochberg) changes in the overall DNA methylation of 273 CpGs (Fig. 1a, b, Supplementary Fig. S1) and correlated changes as described above (Fig. 1c). The principal component analysis (PCA) showed distinct visit-dependent changes mainly due to PC1 96.7% (Fig. 1d). Of note, the top genes, such as *IFI44L* (cg27315157), *MEGF6* (cg06353486), *MX1* (cg08080029), and *CHD5* (cg26312951), contributed to 3.2% of the PC2 distribution (Supplementary Table S2). Using the Illumina HumanMethylation450 annotation data (hg19), we analyzed the corresponding genes as well as the CGI and genomic features of each of the 273 CpG sites (Supplementary Fig. S2A). A statistically significant change in DNA methylation was observed in a time-dependent manner, represented here only for visit 5 and visit 1, in the genomic regions (Supplementary Fig. S2B-C), *viz*. the 5′UTR (adj. *p* value = 0.0007), gene body (adj. *p* value = 8.81e–17; probably due to the maximum number of CpG sites included in this group, *n* = 144 loci) and 3′UTR regions (adj. *p* value = 0.012).

**Fig. 1:**
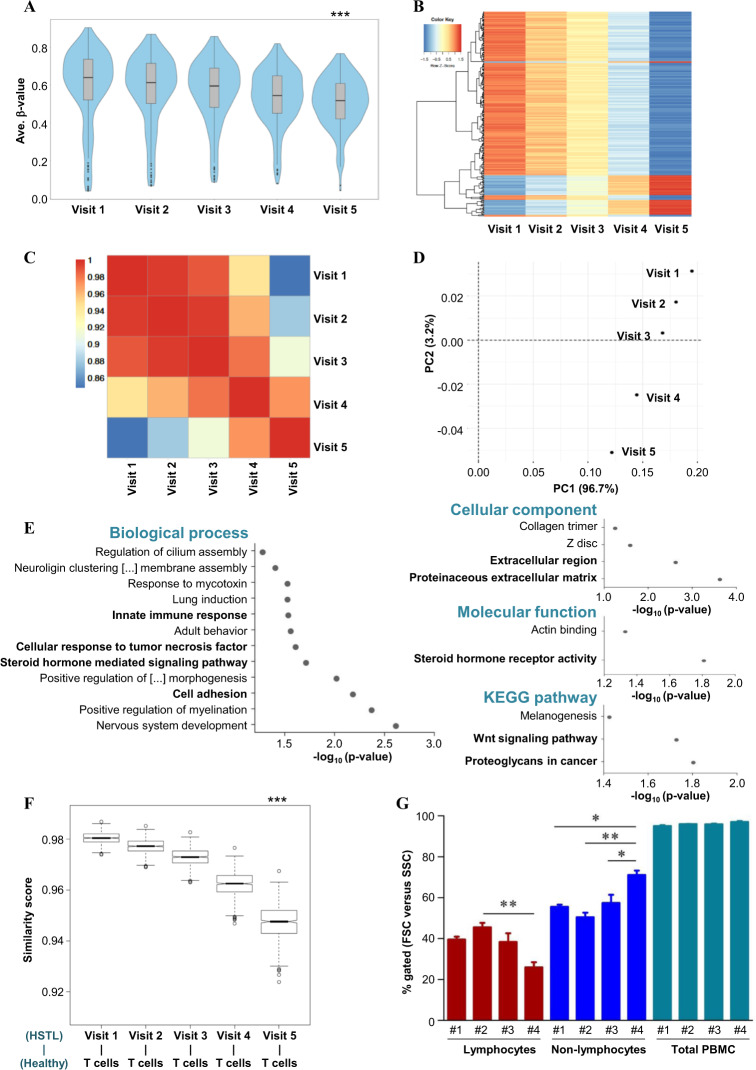
DNA methylation analysis of the γδ-HSTL patient during IFNα2c therapy. DNA methylation analysis was performed on bisulfite-converted DNA from PBMCs of the γδ-HSTL patient obtained at five different time points during continued IFNα2c therapy. **a** The violin plot represents the distribution of the methylation of 273 statistically significant CpG loci at different visits, including the quartiles and median methylation. The DNA methylation level was calculated using the average β-value of the respective CpG site. Only for statistical purposes was the adjusted *p* value calculated for visits 1 and 5 using the paired Wilcoxon test. The statistical significance is shown as *** for *p* values ≤ 0.005. **b** The heatmap was plotted after normalization to the mean methylation level (average β-value) of CpG sites, as indicated by scales in the color key. **c** The heatmap presents Pearson’s correlation values from visit 1 to visit 5. The range of correlation values is shown in the color key at the top-left side of the plot. **d** The principal component analysis (PCA) plot of significant CpG loci (similar to the violin plot) represents the separation of two different components depending on the DNA methylome during the visits of the γδ-HSTL patient. **e** The functional gene ontology (GO) analysis of genes related to the CpG loci from visits 1 to 5. The GO terms enriched in categories, namely, biological process (left-side), cellular component (right-side top), molecular function (right-side middle), and KEGG pathway (right-side lower), are presented for criteria of statistical significance *p* value ≤ 0.05. **f** The cosine similarity score was calculated as described in the Supplementary material. Cosine similarity between the DNA methylation of healthy T cells and cells from the γδ-HSTL patient at visits (1–5) during IFNα2c therapy. The statistical significance of *p* values ≤ 0.05 is represented as *** for the difference between visit 1 and healthy T cells. **g** As shown in Table S1 and represented in Supplementary Fig. 4B, flow cytometry analysis of PBMCs in γδ-HSTL patient visits during IFNα2c therapy was performed. Based on forward scatter (FSC) and sideward scatter (SSC), lymphocytes and non–lymphocytes were gated within the PBMCs. The data are represented as the percentage gated with a statistically significant difference for *p* values ≤ 0.05, ≤0.01, and ≤0.005 shown as ***, **, and *, respectively

Interestingly, the functional annotation of genes related to the 273 CpG sites was enriched for important pathways, such as proteoglycans in cancer (e.g., *CDKN1A*)^8^ and the Wnt signaling pathway, and their regulatory transcription factors (Supplementary Fig. S3A), such as MZF1, SP1, and ARNT, and regulating biological processes, such as steroid hormone signaling and innate immune response (Fig. 1e, Supplementary Table S3), indeed reflect a possible relation of the multiple epigenetic changes with the described clinical features. In addition to the 273 loci retained from the data analysis detailed above, the DNA methylation level of cg26960322 (related to the 5′UTR region of the *CXCR7* gene; an immunologically important gene as well as a potential target for cancer therapy, as it modulates cellular proliferation, survival and migration in the bone marrow and lymphoid organs)^9^ and the WBC counts at the different time points correlated well (*p* value = 0.0167), suggesting a possible functional relevance of regulatory changes in DNA methylation in this γδ-HSTL patient during IFNα2c therapy (Supplementary Fig. S4A).

In a previous report, using the same material (data from visit 1 only) from this γδ-HSTL patient (sample “1_Kab17”), the DNA methylation patterns of HSTL patients, including both αβ- and γδ-HSTL, were compared to those of healthy individuals’ αβ- and γδ-T cells. In this study, altered DNA methylation was observed in HSTL patients exhibiting the hypermethylation of key genes at the active promoter and regulatory enhancer regions.^7^ The γδ-HSTL patient studied in our present longitudinal study carried a STAT3 mutation^7^; thus, IFNα2c can induce proapoptotic gene expression.^10^ However, clonal γδ T cells at visit 1 were refractory to the in vitro treatment of IFNα and lacked proliferative and cytotoxic capacity.^5^ Since the γδ T-cell phenotype of PBMC blasts of this γδ-HSTL patient remained the same (87–93% of lymphocytes), with a steady decrease in WBC/blast counts leading to complete clinical remission after IFNα2c therapy,^5,6^ we used a cosine similarity score model to evaluate the similarity prediction between the DNA methylation profile of the cells from the γδ-HSTL patient as a result of IFNα2c therapy and that of healthy T cells. This computational approach (Fig. 1f), in support of flow cytometry analysis (Fig. 1g, Supplementary Fig. S4B) and clinical data (Supplementary Table S1), revealed that the DNA methylation profile of this patient in response to IFNα2c treatment (leading to clinical remission) was mainly associated with the non–lymphoid compartment of PBMCs and/or IFNα2c-responsive blasts. This observation was also supported by opposing DNA methylation levels of visit 5 and healthy T cells (Supplementary Fig. S3B). Though it is of great interest, it remains still unclear at this point what could be the functional epigenetic status of PBMCs after complete remission is reached, i.e., 6 years after the initiation of therapy.

DNA methylation profiling of the γδ-HSTL patient during the course of IFNα2c therapy in such a comprehensive but in-depth setting has shed light on important biological aspects in terms of (i) the protein regulation of the extracellular matrix and its relevance to cancer progression (e.g., *ADAMTS16*),^11^ (ii) the direct effect of IFNα2c on interferon hallmark gene regulation (e.g., *CDKN1A*, *MX1*),^12^ (iii) the effect of steroids used at the beginning of IFNα2c therapy and the involvement of nuclear receptors important for diverse metabolic functions (e.g., *NR1I3*),^13–15^ and (iv) the change in the (non–)lymphoid compartment in the PBMCs of the γδ-HSTL patient (e.g., *LCN2*),^16,17^ as shown before for the effect of steroids.^18^ To the best of our knowledge, this is the first case report exploring the effect of IFNα2c therapy on DNA methylation and possibly other regulatory epigenetic elements in γδ-HSTL. Based on this retrospective study of a single γδ-HSTL patient, we propose a complex remodeling of the epigenome during IFNα2c therapy, which achieves remission and a clinically healthy state. Though the results of this case report need to be verified in more patients, we provide an in-depth analysis of the IFNα2c therapy-induced changes in CpG methylation, which might serve as potential biomarkers of drug responses and/or disease progression.

## Supplementary information

Supplemental Material

Supplemental Table S1

Supplemental Table S2

Supplemental Table S3
