# Supplementary material for: DNA methylation profile of a hepatosplenic gamma/delta T-cell lymphoma patient associated with response to interferon-α therapy
Source: Cell Mol Immunol. 2020 Aug 20;18(5):1332–5. doi: 10.1038/s41423-020-0518-4 (PMC8093208; doi:10.1038/s41423-020-0518-4)

Supplementary information

DNA methylation profile of a hepatosplenic gamma/delta T-cell lymphoma patient associated with response to Interferon-α therapy

Jaydeep Bhat, Anke K. Bergmann, Silvio Waschina, Christoph Nerl, Christoph Kaleta, Reiner Siebert, Ole Ammerpohl, Dieter Kabelitz

Materials and Methods

Blood samples: Heparinized blood was drawn at the time of clinical inspections with the consent of the patient. The study was carried out in agreement with the declaration of Helsinki. The patient had agreed to use the blood samples for research purpose. At the time of blood collection, institutional review boards were not established. However, a confirmation by the attending physician is available on request (see ref.[1-3]). PBMC were isolated using Ficoll-Hypaque density gradient centrifugation and stored in liquid nitrogen until further use.

Bisulfite conversion of DNA and array hybridization. Genomic DNA was extracted from PBMC of the γδ-HSTL patient using the commercially available Gentra purification kit (Qiagen) following manufacturer's protocol. The quality and quantity of DNA was determined by NanoDrop ND-1000 spectrophotometer (Thermo Fisher Scientific) and Qubit Fluorometer using the Qubit dsDNA BR Assay kit (Life Technologies). Bisulfite conversion of 1 µg of DNA from each sample was performed using EZ-DNA Methylation kit (Zymo Research) according to the protocol provided by the manufacturer. DNA methylation analysis was performed by sample hybridization to the Infinium HumanMethylation450 BeadChip array (also known as '450k arrays'; Illumina), which covers 99 % of RefSeq genes and 96 % of CpG islands. Arrays were scanned using the Illumina iScan[4].

DNA methylation and downstream analysis. Raw hybridization signals were processed using GenomeStudio software (v2011.1; Illumina) in methylation analysis module (v1.9.0; Illumina), applying default settings. The intrinsic controls present on the array were used for data normalization. To exclude technical and biological biases, we used several filters including Y chromosome-specific CpG and 'rs-tags' non-CpG probes. As analyzed by 450k arrays, we

further excluded polymorphisms with an allele frequency > 5 % in the population, which are located within a distance of 0-3 bp next to cytosine. A detection p-value below 0.001 per CpG site was set as internal quality control criteria considering the technical results of hybridization. CpG sites were further annotated to HumanMethylation450 v1.2 Manifest file (Illumina) annotating the UCSC Genome database (hg19 version)[5].

The methylation level at each CpG is represented by the average β-value, defined as the proportion of the methylated signal to the total signal and calculated from normalized intensity values. Statistical analysis was performed on the list of CpG sites passing above mentioned criteria using Qlucore Omics Explorer (v2.3(45), 64-bit; Qlucore) software. We used unsupervised way to filter by variance ($\sigma/\sigma_{max} > 0.1$). This approach allows us to remove variables which are almost constant across all the observations. In this study, only one sample per group was investigated for global DNA methylation. The location of CpG sites in relation to the genomic region with the possibility of alternative TSS and regions containing more than one gene were defined as follow: TSS1500 (from 201 to 1500 bp upstream of the TSS), TSS200 (from 1 to 200 bp upstream of the TSS), 5' UTR, 1st Exon, gene body and 3' UTR. Relation of CpG site to CpG islands (CGI) was defined as CGI (within CpG Islands), CGI shore (0-2 kb from CGI edge), CGI shelf (> 2 kb to 4 kb from CGI edge) and outside CGI, referred here as the open sea[6]. Wherever indicated, adjusted p-values were calculated using non-parametric paired Wilcoxon signed-rank test with multiple hypothesis testing correction using the Benjamini-Hochberg method[7]. Statistical analysis and graphs were generated using R (3.6.1), Bioconductor packages and ggplot2[8]. For the downstream analysis, the functional processes were annotated using functional annotation tools such as DAVID[9], Enrichr[10, 11]. The 450k array data is deposited into the Gene expression omnibus (GEO) with accession number GSE105078.

Cosine-similarity analysis. The cell type similarity score was measured between the DNA methylation profile of IFNα2c-treated PBMC of the γδ-HSTL patient and healthy T cells as described previously[12]. Briefly, mean DNA methylation was calculated for each CpG loci in each cell type. Then similarity score for two different cell types was calculated by adding cosine of these two cell types of interest. This score was normalized to the range of 0 and 1. We used 266 CpG sites, which were common both in the γδ-HSTL patient and healthy T cells, to randomly resample 100,000 times using a bootstrapping approach and then the statistical difference was calculated using the Wilcoxon signed-rank for the comparison. The resulting

similarity score for cell types, i.e. visit 1-5 and healthy T cells, is presented in the box plot. The code used to calculate the similarity score will be deposited in the GitHub repository https://github.com/JaydeepBhat

Protein expression measured by flow cytometry. Cell surface expression was analyzed by flow cytometry following standard protocols[13]. Briefly, thawed PBMC were washed once with staining buffer and then incubated for 25 mins on ice with diluted phycoerythrin (PE)-conjugated antibody against human CXCR7 (clone 8F-11-M16; Cat # 331103; BioLegend) or isotype control mouse IgG2b (clone 133303; Cat # IC0041P; R&D Systems). Then samples were washed once with staining buffer and suspended in 1% paraformaldehyde. Samples were measured on a BD FACSCalibur flow cytometer (BD Biosciences). Data analysis was done using the FlowJo software (FlowJo LLC, Ashland Or, USA). The correlation analysis of CXCR7 protein expression, DNA methylation level and leukocyte count was done and graphically represented by using GraphPad Prism version 6.0 for Windows, GraphPad software.

Legends to Supplementary Figures:

Supplementary Figure S1: CpG methylation associated changes in the IFNα2c-treated γδ-HSTL patient. As described in the legend of figure 1, DNA methylation was performed on PBMC from the γδ-HSTL patient during IFNα2c treatment. At five different time points labeled as visit 1-5, the heatmap is plotted without normalizing to the mean methylation level (average β-value) of CpG sites as indicated by scales in the color key.

Supplementary Figure S2: γδ-HSTL patient-associated CpG sites annotated to the UCSC. Statistically significant CpG loci associated with the γδ-HSTL patient during IFNα2c treatment were annotated to the UCSC Genome database (hg19 version) as described in the supplementary methods. (A) The distribution of CpG loci according to the CpG Island features (left-side) and genomic features (right-side) is presented in the histogram. (B) The methylation levels based on the average β-value of CpG loci are represented at the respective genomic elements. Excluding non-annotated CpG loci, all the significant loci were used for the calculation and the median methylation of the group/visit of the γδ-HSTL patient were connected by straight line using ggplot2 package in the R-program. The adjusted p-value was calculated between each genomic

feature using a paired Wilcox test and shown by *** for ≤ 0.005 and ** for ≤ 0.001. (C) The change in the average β-value of CpG loci of the γδ-HSTL patient according to the visits is separately represented for TSS1500 (upper left), TSS200 (middle left), 5' UTR (lower left), 1st Exon (upper right), Gene body (middle right), 3' UTR (lower right) using violin plot.

Supplementary Figure S3: Functional gene ontology (GO) analysis of a IFNα2c-treated γδ-HSTL patient. (A) As described in Fig. 1E, genes annotated to the significant CpG sites of IFNα2c-treated γδ-HSTL patient are found to be regulated by listed transcription factors using Enrichr-based JASPAR and TRANSFAC. Transcription factors with enrichment of statistically significant p-value ≤ 0.05 are considered for the representation. (B) The methylation levels based on the average β-value of CpG loci of the γδ-HSTL patient during the respective visits and of healthy T cells is plotted as a heatmap. The normalization of CpG sites across the sample is performed and represented as per the color key.

Supplementary Figure S4: Correlation analysis of CpG methylation, protein expression and leukocyte counts. As described in the supplemental information, ficoll-separated PBMC from the γδ-HSTL patient at four different time-points (i.e. 0 day/at diagnosis: #1, 155 days/visit 2: #2, 253 days/visit 3: #3 and 652 days/visit 5: #4) were subjected to flow cytometry analysis. (A) The peripheral blood leukocyte counts of the γδ-HSTL patient at three different time-points was correlated with the respective leukocyte counts, the average β-value of cg26960322 site measured by the DNA methylation 450k array and the mean fluorescence intensity (MFI) of CXCR7 cell surface expression measured by flow cytometry. The Pearson's correlation coefficient r was considered between – 1 and + 1, where – 1 represents total negative correlation and + 1 represents total positive correlation between two indicated variables. The coefficient of determination is shown by r^2 value, which ranges between 0 and 1. A p-value ≤ 0.05 is considered statistically significant. Abbreviations: Meth, methylation level (average β-value); Prot, protein expression level (mean fluorescence intensity); Leuk, Leukocyte count. (B) As summarized in the figure 1G, the dot plot representing FSC versus SSC are shown. The populations were gated based on forward scatter (FSC) and side scatter (SSC) into lymphocytes and monocytes (i.e., non-lymphocytes) in the dot plot. Samples were acquired on BD Calibur and the data was analyzed using FlowJo software.

Legends to Supplementary Tables:

Supplementary Table S1: Clinical information on γδ-HSTL patient. The information about WBC counts and composition, course of therapy, pathological conditions are described in the table. The analysis performed or not is indicated by + or –, respectively. Abbreviations used: N/A, Not Available; Hb, Hemoglobin; IFN-a2c, Interferon-a2c; PEG-A, Pegylated Interferon-a2c; +, analysis performed in this study; –, analysis not performed in this study.

Supplementary Table S2: List of statistically significant CpG sites. The PBMC from each visit of γδ-HSTL patient were subjected to analysis of DNA methylation as described in supplementary methods and in the main text. A list of 273 CpG loci correlated with days treatment and methylation level was annotated to the UCSC genome database (hg19). The CpG loci in the respective genes are listed in the separate tabs of the table. The quantitative contribution of scores of individual factors (i.e. "TargetID" or "CpG loci") are listed as per the PCA axes PC1 and PC2.

Supplementary Table S3: Correlation analysis of the time interval of the IFNα2c therapy and the respective CpG methylation level. The GO enrichment analysis of 273 CpG sites of γδ-HSTL patient visit during the treatment of IFNα2c therapy were annotated using DAVID web-based tool. The GO terms according to the biological process, cellular component, molecular function, KEGG pathway and reactome pathway are listed with their details including p-value and genes.

Supplementary references:

1. Metzger R, Heckl-Ostreicher B, Nerl C, Schondelmaier S, Kabelitz D. Immunological studies of gamma delta T cells in a case of large granular lymphocyte (LGL) leukemia: leukemic gamma delta+ T cells are resistant to growth stimulation in vitro but respond to interferon-alpha treatment in vivo. Leukemia research. **16**:1087-95 (1992).
2. Hinz T, Marx S, Nerl C, Kabelitz D. Clonal expansion of gamma delta T cells expressing two distinct T-cell receptors. British journal of haematology. **94**:62-4 (1996).

3. Bergmann AK, Fataccioli V, Castellano G, Martin-Garcia N, Pelletier L, Ammerpohl O, et al. DNA methylation profiling identifies candidate genes for the pathogenesis of hepatosplenic T-cell lymphoma. Haematologica. **104**:e104-e7 (2019).

4. Bibikova M, Barnes B, Tsan C, Ho V, Klotzle B, Le JM, et al. High density DNA methylation array with single CpG site resolution. Genomics. **98**:288-95 (2011).

5. Kent WJ, Sugnet CW, Furey TS, Roskin KM, Pringle TH, Zahler AM, et al. The human genome browser at UCSC. Genome research. **12**:996-1006 (2002).

6. Kulis M, Merkel A, Heath S, Queiros AC, Schuyler RP, Castellano G, et al. Whole-genome fingerprint of the DNA methylome during human B cell differentiation. Nature genetics. **47**:746-56 (2015).

7. Benjamini Y, Hochberg Y. Controlling the False Discovery Rate: A Practical and Powerful Approach to Multiple Testing. Journal of the Royal Statistical Society Series B (Methodological). **57**:289-300 (1995).

8. Wickham H. ggplot2: Elegant Graphics for Data Analysis (2009).

9. Huang da W, Sherman BT, Lempicki RA. Systematic and integrative analysis of large gene lists using DAVID bioinformatics resources. Nature protocols. **4**:44-57 (2009).

10. Chen EY, Tan CM, Kou Y, Duan Q, Wang Z, Meirelles GV, et al. Enrichr: interactive and collaborative HTML5 gene list enrichment analysis tool. BMC bioinformatics. **14**:128 (2013).

11. Kuleshov MV, Jones MR, Rouillard AD, Fernandez NF, Duan Q, Wang Z, et al. Enrichr: a comprehensive gene set enrichment analysis web server 2016 update. Nucleic acids research. **44**:W90-7 (2016).

12. Durek P, Nordstrom K, Gasparoni G, Salhab A, Kressler C, de Almeida M, et al. Epigenomic Profiling of Human CD4+ T Cells Supports a Linear Differentiation Model and Highlights Molecular Regulators of Memory Development. Immunity. **45**:1148-61 (2016).

13. Chitadze G, Fluh C, Quabius ES, Freitag-Wolf S, Peters C, Lettau M, et al. In-depth immunophenotyping of patients with glioblastoma multiforme: Impact of steroid treatment. Oncoimmunology. **6**:e1358839 (2017).

Supplementary figure 1

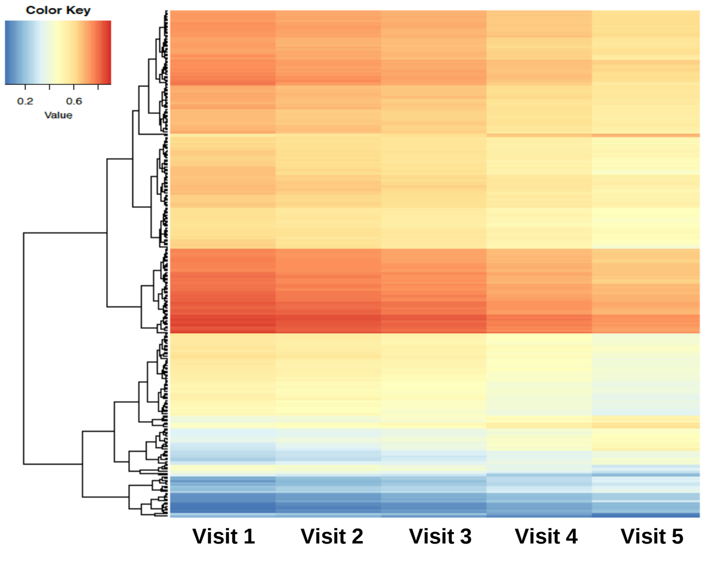

Supplementary figure 2

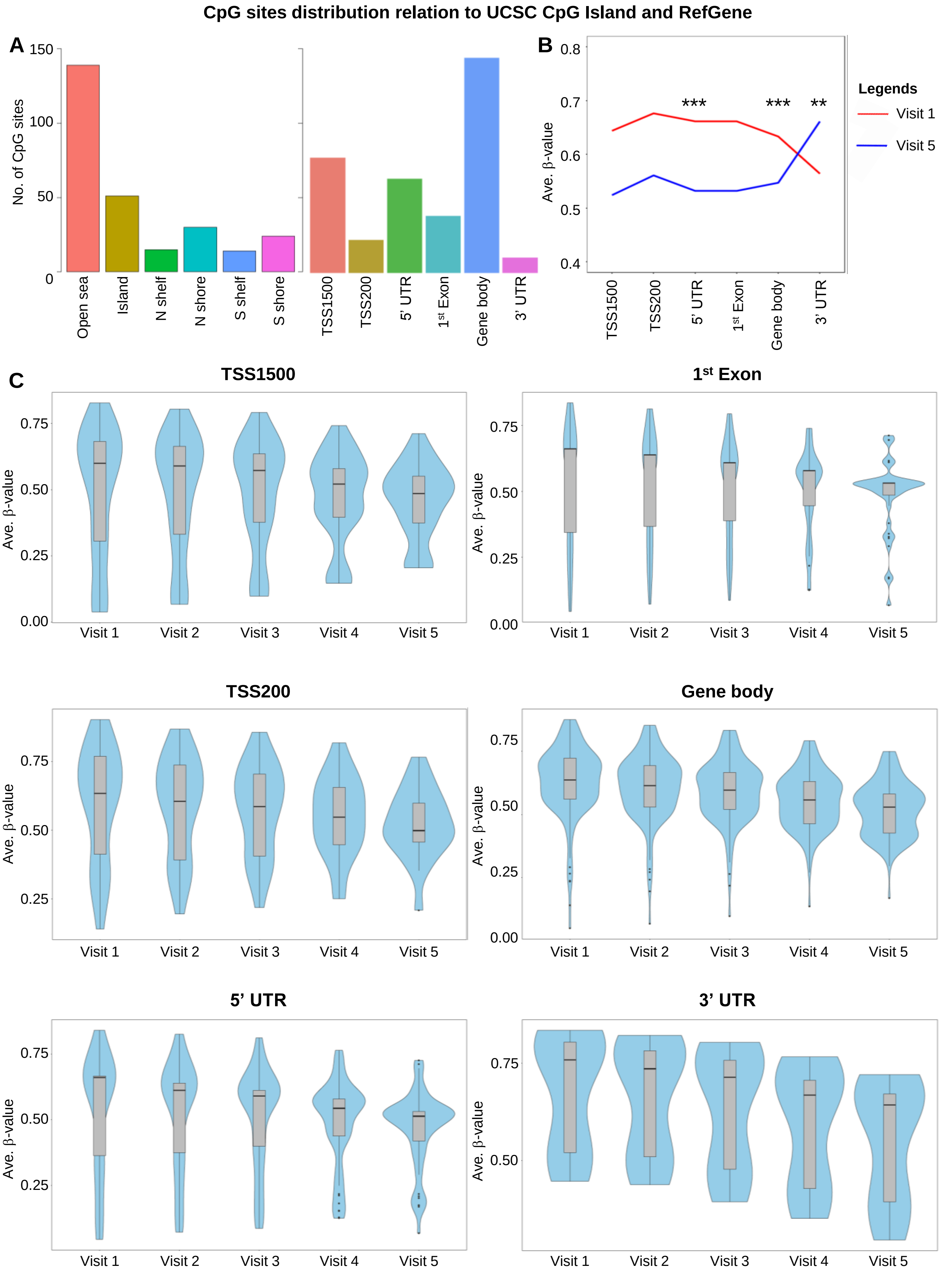

Supplementary figure 3

A

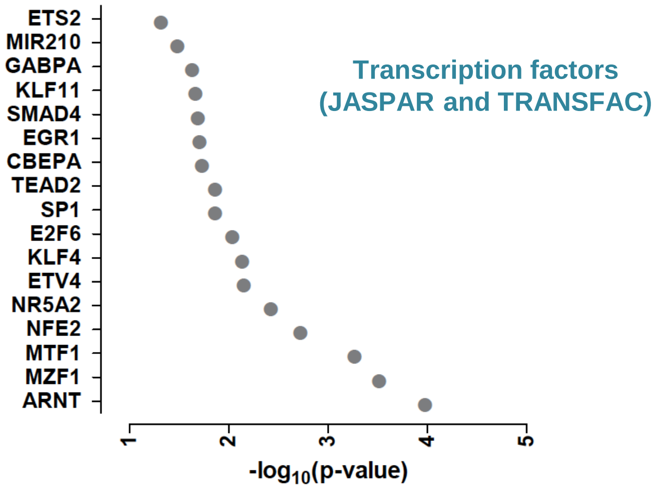

B

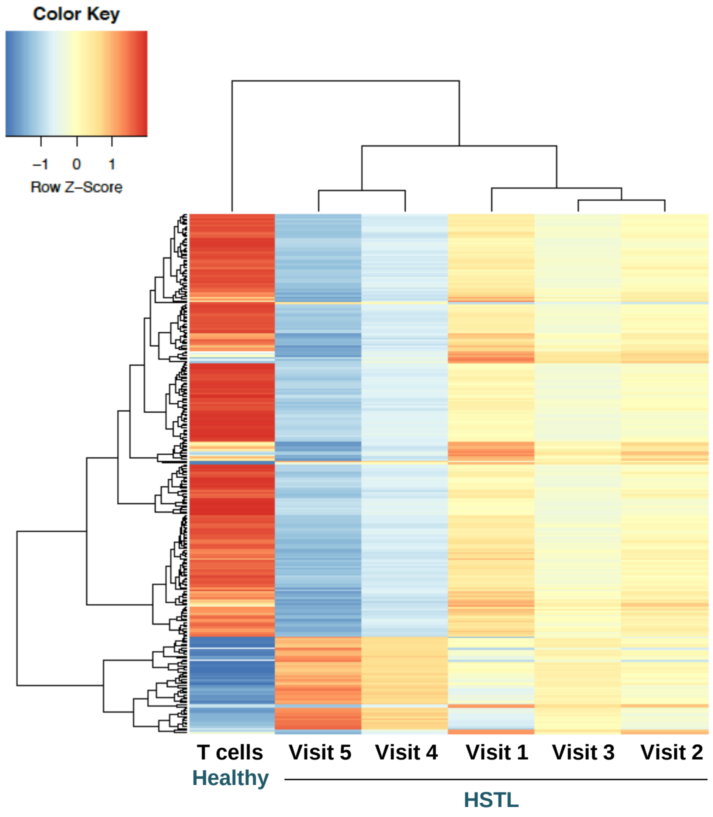

Supplementary figure 4

A

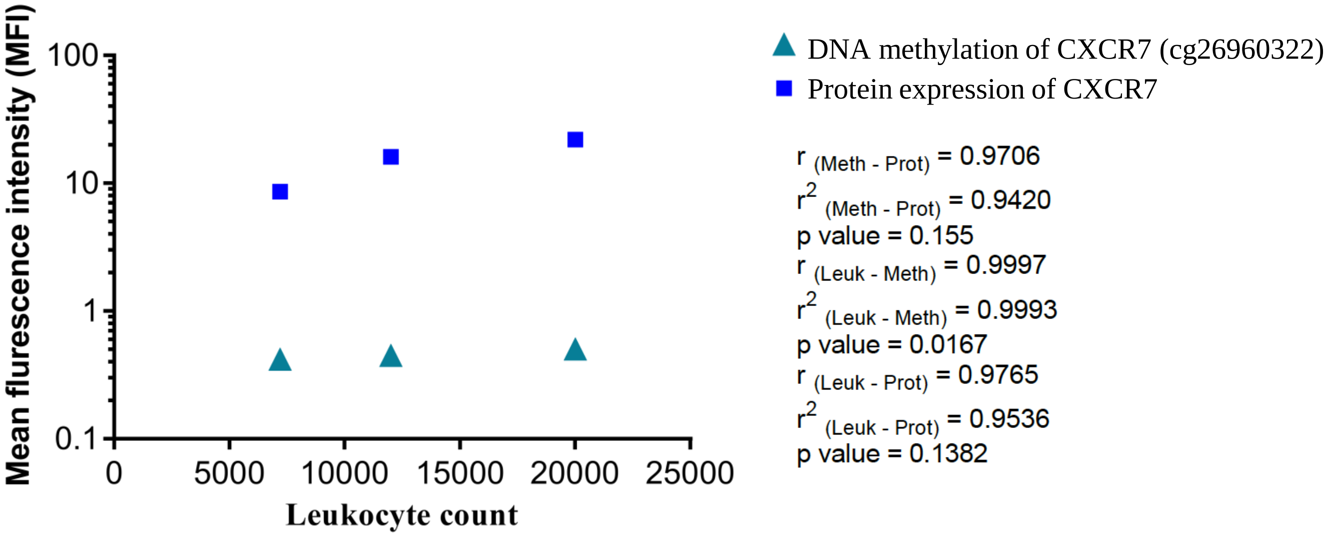

B

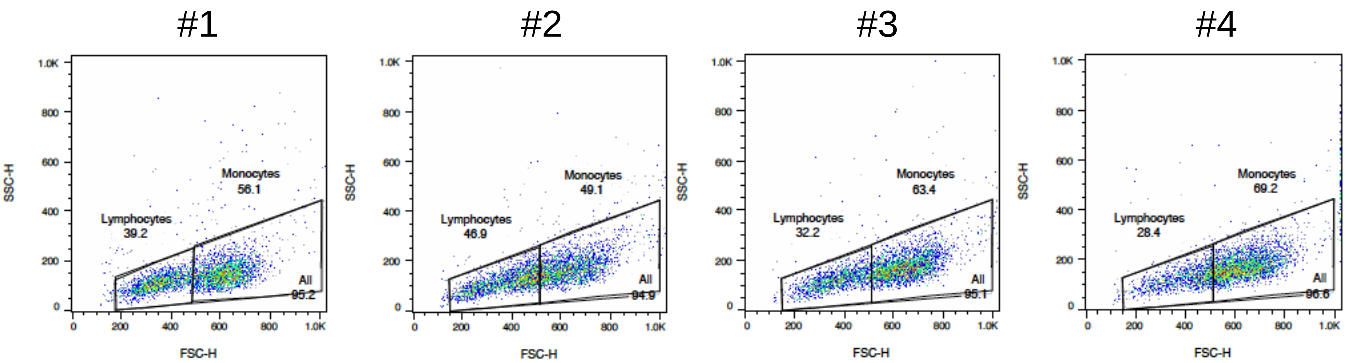

Supplement: Supplementary file 1 — Supplemental Material [file 41423_2020_518_MOESM1_ESM.pdf]
